# Ecological Speciation Promoted by Divergent Regulation of Functional Genes Within African Cichlid Fishes

**DOI:** 10.1093/molbev/msac251

**Published:** 2022-11-15

**Authors:** Madeleine Carruthers, Duncan E Edgley, Andrew D Saxon, Nestory P Gabagambi, Asilatu Shechonge, Eric A Miska, Richard Durbin, Jon R Bridle, George F Turner, Martin J Genner

**Affiliations:** School of Biological Sciences, University of Bristol, Bristol BS8 1TQ, United Kingdom; School of Biological Sciences, University of Bristol, Bristol BS8 1TQ, United Kingdom; School of Biological Sciences, University of Bristol, Bristol BS8 1TQ, United Kingdom; Tanzanian Fisheries Research Institute, Kyela Research Centre, P.O. Box 98, Kyela, Mbeya, Tanzania; Tanzanian Fisheries Research Institute, Dar es Salaam Research Centre, P.O. Box 9750, Dar es Salaam, Tanzania; Wellcome/CRUK Gurdon Institute, University of Cambridge, Cambridge CB2 1QN, United Kingdom; Department of Genetics, University of Cambridge, Cambridge CB2 3EH, United Kingdom; Wellcome Sanger Institute, Wellcome Genome Campus, Cambridge CB10 1SA, United Kingdom; Department of Genetics, University of Cambridge, Cambridge CB2 3EH, United Kingdom; Wellcome Sanger Institute, Wellcome Genome Campus, Cambridge CB10 1SA, United Kingdom; School of Biological Sciences, University of Bristol, Bristol BS8 1TQ, United Kingdom; School of Natural Sciences, Bangor University, Bangor, Wales LL57 2UW, United Kingdom; School of Biological Sciences, University of Bristol, Bristol BS8 1TQ, United Kingdom

**Keywords:** molecular evolution, ecological speciation, gene expression, transcriptional regulation, cichlids

## Abstract

Rapid ecological speciation along depth gradients has taken place repeatedly in freshwater fishes, yet molecular mechanisms facilitating such diversification are typically unclear. In Lake Masoko, an African crater lake, the cichlid *Astatotilapia calliptera* has diverged into shallow-littoral and deep-benthic ecomorphs with strikingly different jaw structures within the last 1,000 years. Using genome-wide transcriptome data, we explore two major regulatory transcriptional mechanisms, expression and splicing-QTL variants, and examine their contributions to differential gene expression underpinning functional phenotypes. We identified 7,550 genes with significant differential expression between ecomorphs, of which 5.4% were regulated by *cis*-regulatory expression QTLs, and 9.2% were regulated by *cis*-regulatory splicing QTLs. We also found strong signals of divergent selection on differentially expressed genes associated with craniofacial development. These results suggest that large-scale transcriptome modification plays an important role during early-stage speciation. We conclude that regulatory variants are important targets of selection driving ecologically relevant divergence in gene expression during adaptive diversification.

## Introduction

Ecological opportunity has the ability to enable ecological speciation and large-scale phenotypic diversification (Schluter 2001, 2009; Rundle and Nosil 2005). Commonly, populations diverge in response to different selective pressures along ecological gradients, and understanding the dynamics of such organism–environment interactions is a fundamental goal in eco-evolutionary research (Doebeli and Dieckmann 2002; Schluter and Pennell 2017). In freshwater fishes, ecological diversification along the habitat-depth gradient into contrasting trophic ecomorphs has taken place independently and repeatedly (Schluter 1996; Sturmbauer 1998; Jonsson and Jonsson 2001; Seehausen 2006; Bernatchez et al. 2010; Rundle and Schluter 2012), providing powerful natural systems to study mechanisms of ecological speciation. Here we focus on cichlid fishes, which represent valuable candidates for such investigations given their widespread ecological speciation and adaptive radiation (Sturmbauer 1998; Seehausen 2006). However, while their phenotypic diversity has been well documented, and substantial insights into the genetics of population divergence in these systems have been achieved in recent years (Sturmbauer 1998; Seehausen 2006; Joyce et al. 2011; Salzburger 2018), the molecular basis and mechanisms facilitating such rapid, ecologically driven diversification are less well understood.

The transcriptome links the genotype and phenotype and is shaped by both genetic and environmental factors (Pavey et al. 2010), offering a fundamental tool in our understanding of how environmental variation and its effects on gene expression affect local adaptation and speciation (Ranz and Machado 2006; Rockman and Kruglyak 2006). Recent genome-wide studies have revealed widespread variation in gene expression among populations undergoing adaptation to ecologically divergent environments (Abzhanov et al. 2006; Meng et al. 2013; Zhao et al. 2015; Singh et al. 2017; Verta and Jones 2019; Jacobs and Elmer 2021). In addition, variation in gene expression and regulation has been shown to have a substantial heritable component upon which selection can act (Skelly et al. 2009; Leder et al. 2015). Therefore, genetic variants regulating gene expression are likely to play a major role in facilitating adaptive diversification, and ultimately ecological speciation.

**Fig. 1.**
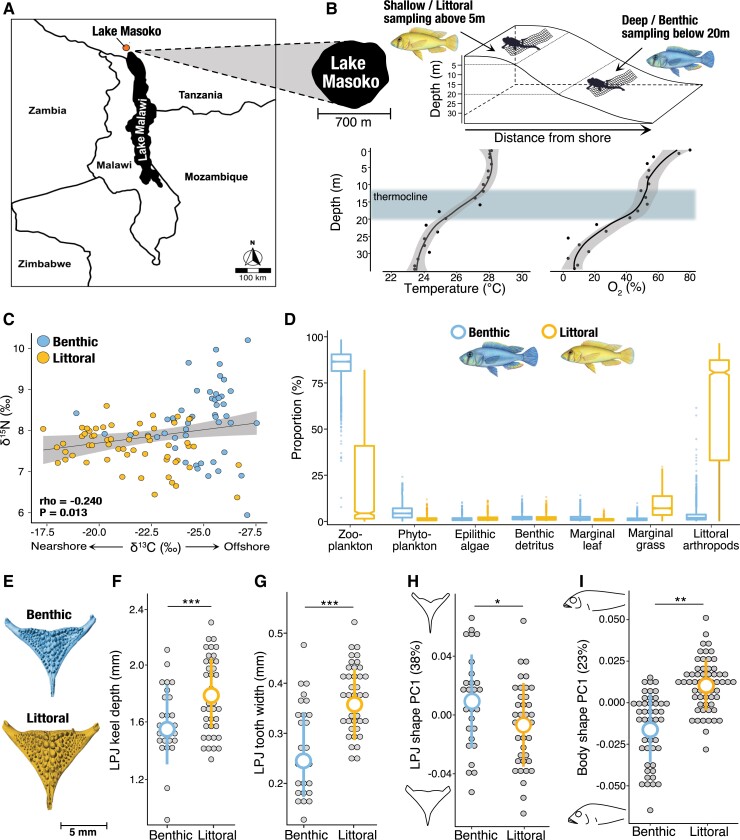
Sampling information and phenotypic divergence. (*A*) The location of Lake Masoko, relative to Lake Malawi and bordering countries. (*B*) Schematic of sampling approach for collection of shallow-water littoral and deep-water benthic ecomorphs of *A. calliptera* in Lake Masoko, as well as temperature and oxygen profiles by depth gradient (data from Delalande 2008). (*C*) Association between carbon and nitrogen stable isotope signatures, demonstrating differences in trophic feeding regimes between benthic and littoral ecomorphs (n = 113). (*D*) Results from Bayesian stable isotope mixing models showing proportional estimates (mean, 25% and 75% percentiles) of diet composition for benthic and littoral ecomorphs. (*E*) Example of LPJ images segmented from micro-CT X-ray scans of craniofacial morphology, showing a representative example for benthic (top image) and littoral (bottom image) ecomorphs. (*F–I*) Differences in LJP keel depth (n = 70), LPJ tooth width (*n* = 70), LPJ shape (*n* = 70), and body shape (*n* = 113), respectively, between benthic and littoral ecomorphs. Grey points represent individual samples, with mean ± SD values for each ecomorph represented by the larger coloured points and error bars (benthic, blue; littoral, yellow). LPJ shape and body shape change are shown along the first principal component axis (PC1). The proportion of variation explained is given in parentheses. LPJ and body shape outlines represent shapes at axis extremes along PC1. Number of asterisks represents the level of significance (* < 0.05, ** < 0.01, *** < 0.001).

To understand how divergent gene expression and regulation might promote ecological diversification in nature, we generated whole transcriptome data from a population of cichlid fishes currently undergoing the early stages of sympatric speciation. In the East African crater lake, Lake Masoko (also known locally as Kisiba), *Astatotilapia calliptera* has diverged along a depth gradient into shallow-water “littoral” and deep-water “benthic” ecomorphs within the last 1,000 years (∼500 generations) (Malinsky et al. 2015). The littoral ecomorph inhabits the warm, well lit, highly oxygenated surface waters (above 5 month), whereas the benthic ecomorph inhabits the cool, poorly lit, oxygen depleted, deep-water (below 20 month) environment in Lake Masoko (fig. 1; Delalande 2008). The extent of phenotypic and genomic divergence between these ecomorphs has been well characterized (Malinsky et al. 2015), revealing that despite substantial divergence in craniofacial morphology and feeding ecology, just a small number of genomic variants show equivalently strong divergence. The key issue, therefore, is to understand the molecular mechanisms that generate such dramatic divergent shifts in phenotype in the absence of widespread and extensive genomic differences.

Here we explore two major heritable mechanisms of transcriptional regulation and modification, expression-quantitative trait loci (eQTLs) and splicing-QTL (sQTLs), to elucidate the molecular basis of ecological speciation. Since transcription has substantial genetic control, eQTL, and sQTL mapping provides information about genetic variants with modular effects on gene expression (Carroll et al. 2005; Wittkopp and Kalay 2012; Ishikawa et al. 2017; Verta and Jones 2019; Leal-Gutiérrez et al. 2020; Kerimov et al. 2021; Patro et al. 2021), which are useful for understanding the genomic architecture and evolution of complex traits. Both eQTLs and sQTLs can be classified into *cis* (local) and *trans* (distant) effects. *Cis*-regulatory mutations are thought to be associated with fewer adverse effects than *trans*-regulatory mutations or protein-coding changes and have been strongly implicated in rapid adaptation and diversification, allowing rapid trait divergence while minimizing covariance with other traits (and potentially negative selective effects) (Prud’homme et al. 2006; Ishikawa et al. 2017; Verta and Jones 2019). Therefore, here we investigated variation in *cis*-acting eQTLs and sQTLs between the Lake Masoko ecomorphs to understand how transcriptional regulation facilitates divergence in functionally relevant traits during the initial stages of speciation. We predict that phenotypic diversity associated with trophic niche divergence will be facilitated by a combination of transcriptional modification from *cis*-acting expression and splicing QTLs.

In this study, we first describe the extent of ecological (trophic diet) and phenotypic (body and jaw morphology) diversity between the benthic and littoral *A. calliptera* ecomorphs of Lake Masoko, with specific focus on a highly adaptive cichlid trait, the lower pharyngeal jaw (LPJ), which is associated with divergence in trophic diet (Rüber and Adams 2001; Muschick et al. 2012; Gunter et al. 2013; Kusche et al. 2014; Burress 2016). Secondly, using whole transcriptome data from LPJ tissue, we investigate *cis*-acting expression and splicing QTLs to characterize the genetic architecture of regulatory variation and quantify the relative contribution of these mechanisms to divergent gene expression. Thirdly, we identify whether ecomorph-specific patterns of gene expression and regulatory variation are associated with signatures of recent selection.

## Results

### Evidence for Adaptive Trophic Divergence in Eco-morphological Traits

We measured eco-morphological traits linked to trophic divergence in cichlids, namely body morphology, and LPJ morphology (Rüber and Adams 2001; Muschick et al. 2012; Kusche et al. 2014; Burress 2016) (*n* = 113; supplementary table S1, Supplementary Material online) and identified differences in feeding regimes between the two ecomorphs using carbon and nitrogen stable isotope ratios. Littoral individuals have lower δ^13^C values relative to benthics (Kruskal-Wallis: χ^2^ = 48.846, *P* = 2.131e-11; fig. 1*C*), while the benthic fish have higher δ^15^N values relative to littorals (Kruskal-Wallis: χ^2^ = 17.300, *P* = 3.191e-05; fig. 1*C*), indicating differences in trophic food source and trophic level, respectively. Bayesian stable isotope mixing models were used to determine diet compositions for benthic and littoral ecomorphs. These show considerable differences in the contributions of dietary materials to the overall feeding regimes of the ecomorphs (fig. 1*D*; see supplementary table S2, Supplementary Material online for further information of dietary materials). The highest diet source contributor in benthic ecomorphs is zooplankton (85.4 ± 0.079%; mean ± SD), with a small proportion of their diet comprising of phytoplankton (5.1 ± 0.036%; mean ± SD). The diet of littoral ecomorphs is most strongly contributed to by littoral arthropod macroinvertebrates (63.3 ± 0.346%; mean ± SD), with additional contributions from consuming zooplankton and marginal grass (21.8 ± 0.280% and 9.0 ± 0.067%, respectively; mean ± SD).

We also show that the overall morphology of LPJs differs markedly between benthic and littoral ecomorphs (fig. 1*E*). Littoral fish have significantly deeper LPJs (analysis of covariance: *F*_1,63_ = 12.866, *P* = 0.0006; fig. 1*F*), and wider teeth (analysis of covariance: *F*_1,63_ = 28.916, *P* = 1.176e-06; fig. 1*G*) than benthic individuals. Differences in the shape of the LPJ between ecomorphs (multivariate analysis of covariance: Wilk's lambda_1,60_ = 0.572, *P* = 2.052e-06; fig. 1*H*; supplementary fig. S1, Supplementary Material online), are in the relative length and width (affects leveraging power (Muschick et al. 2012; Burress 2016)), the size and position of the lateral processes (important muscle attachment sites (Muschick et al. 2012; Burress 2016)), and the shape of the dentigerous area. Differences in body shape are also clear between benthic and littoral ecomorphs (multivariate analysis of covariance, Wilk's lambda_1,58_ = 0.566, *P* = 2.592e-06; fig. 1*I*; supplementary fig. S2, Supplementary Material online), including body depth, head depth, and length, size and position of the pectoral fin (important for maneuvring (Snorrason et al. 1992; Svanbäck and Eklöv 2002; Olsson and Eklöv 2005)), and size and position of the mouth (associated with feeding modality (Ahi et al. 2019; Hulsey et al. 2020)). Taken together, these results indicate a substantial and consistent shift in feeding behavior and trophic niche specialization between the benthic and littoral ecomorphs.

### Divergent Gene Expression Underlies Adaptive Phenotypes

**Fig. 2.**
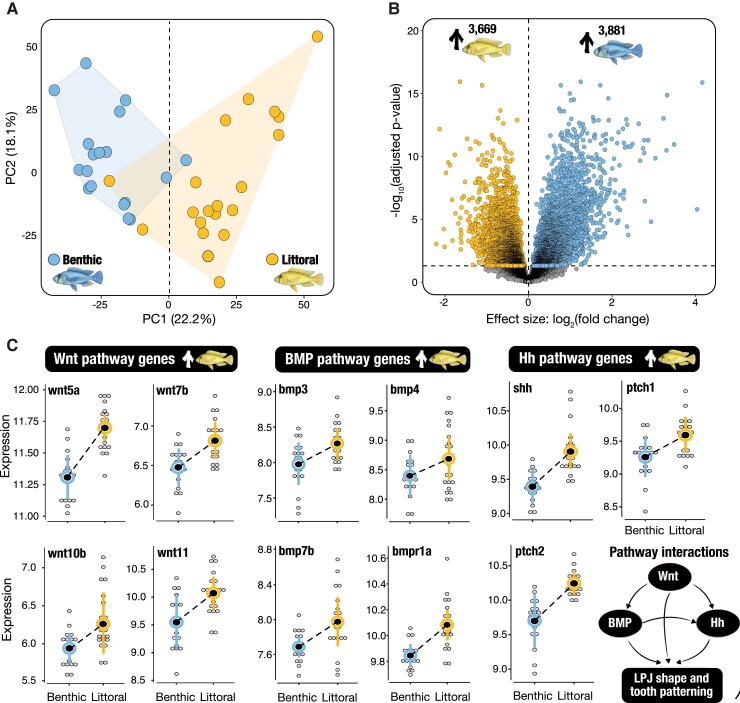
Divergent gene expression. (*A*) Principal component analysis showing variation in gene expression profiles of benthic (blue; *n* = 18 individuals) and littoral (yellow; *n* = 20 individuals) ecomorphs along principal components 1 (PC1) and 2 (PC2). The percent of total variation explained by each principal component axis is given in parentheses. (*B*) Volcano plot highlighting the extent of divergence in gene expression. Significant differentially expressed genes are colored according to the expression direction (yellow, significantly upregulated in littoral; blue, significantly upregulated in benthic; FDR < 0.05). Gene expression analyses are based on the complete set of expressed genes after filtering for low counts (*n* = 19,237 genes). (*C*) Differential expression of “master adaptation genes” involved in three major pathways implicated in LPJ plasticity networks and trophic adaptation (Wnt-signaling, BMP-signaling and Hh signaling). Grey points represent individual samples, with mean ± SD values for each ecomorph represented by the larger colored points and error bars (benthic, blue; littoral, yellow). A schematic of pathway interactions during LPJ shape and tooth development is given in the bottom right.

Genome-wide gene expression was studied using 38 whole transcriptomes from LPJ tissue (18 benthics and 20 littorals), with an average sequencing depth of 37 million reads per library (supplementary table S3, Supplementary Material online). Cleaned reads were mapped against the *Maylandia zebra* reference genome (UMD2a, NCBI assembly: GCF_000238955.4) (Conte et al. 2019), with a mean mapping success of 84% (supplementary table S3, Supplementary Material online). Global expression patterns were initially explored using a Principal Component Analysis (PCA; based on the complete dataset of 19,237 genes, after filtering for low counts). The PCA demonstrates clear differences in the overall expression profiles of benthic and littoral individuals along the primary axis of variation (PC1; fig. 2*A*). A remarkably high proportion of genes (39%) are significantly differentially expressed (DE) (FDR-corrected *P* < 0.05; fig. 2*B*; supplementary table S4, Supplementary Material online), consistent with large regulatory shifts across the transcriptome. Moreover, we found that both the proportion and magnitude (extent of expression divergence measured as log_2_ fold change) of DE genes upregulated in one ecomorph relative to the other is consistent (51% of DE genes upregulated in benthics, 49% in littorals; fig. 2*B*). This consistent level of expression modulation across both ecomorphs suggests widespread upregulation and downregulation have facilitated phenotypic divergence in Masoko (Malinsky et al. 2015).

Next, we investigated transcriptional evidence for functional differences between ecomorphs using functional enrichment analysis of Gene Ontology (GO) biological process annotations associated with DE genes. GO terms enriched in benthic ecomorphs are related to blood cell development, vascular morphogenesis and metabolic oxidation-reduction processes. This may indicate local adaptation to increased habitat depth (Jacobs and Elmer 2021; Vernaz et al. 2022) and, more specifically, to the relatively low oxygen conditions of the deep-water benthic environment in Lake Masoko, compared with shallow-water habitat (fig. 1*B* and supplementary table S5, Supplementary Material online). Interestingly, we also found significant enrichment of several immune response processes. Variation in parasite exposure, and consequently expression of immune gene networks, has been proposed as a major selective pressure and driver of adaptation and evolution in ecologically diversifying populations (Eizaguirre and Lenz 2010; Eizaguirre et al. 2012; Lenz et al. 2013a, b). We therefore conducted an analysis of gill ectoparasites. We found that the gills of both ecomorphs are parasitized by a single species, the planktonic copepod *Lamproglena monodi*. However, as expected given their different habitats, infection rates were significantly higher in benthic (83%, *n* = 15 of 18) compared to littoral (36%, *n* = 9 of 25) individuals (GLM: Z = −2.885, *P* = 0.004; supplementary fig. S3, Supplementary Material online).

GO terms enriched in littoral ecomorphs are involved in several ecologically relevant functions, including calcium pathways, bone remodeling, muscle contraction, and neural development (supplementary table S5, Supplementary Material online). We also found that 86% (73 out of 85) of genes previously implicated in cichlid jaw plasticity networks were differentially expressed between the Lake Masoko species pair (supplementary table S6, Supplementary Material online). Specific genes included several bone morphogenic proteins (BMPs), *bmp3*, *bmp4*, *bmp7b*, and *bmpr1b* (fig. 2*C*), which play key roles in craniofacial development (Wan and Cao 2005; Nie et al. 2006). Moreover, *bmp4* is a known driver of craniofacial divergence during adaptive radiation in cichlid complexes (Streelman and Albertson 2006; Albertson 2008; Parsons and Albertson 2009; Gunter et al. 2013), and Darwin's finches (Abzhanov et al. 2004). In both cases, expression variation in *bmp4* is functionally linked to differences in foraging strategy. We also found consistent significant upregulation of several Wnt (Wingless-related integration site) and Hh (Hedgehog) pathway genes in littoral ecomorphs, including *wnt5a, wnt7b, wnt10b* and *wnt11* and *shh, ptch1* and *ptch2* (fig. 2*C* and Supplementary tables S4 and S6, Supplementary Material online). Given the known importance of Wnt and Hh pathways and their interactions with BMP pathways in LPJ shape and tooth patterning (Gunter et al. 2013; Schneider et al. 2014; Hulsey et al. 2016; Singh et al. 2017, 2021), these results evidence the role of divergent expression regulation in facilitating phenotypic shifts a major adaptive trait, the LPJ.

### Large-effect Expression and Splicing QTLs Regulate Divergent Gene Expression

While gene expression can be highly plastic in its response to environmental cues, divergent expression patterns need to be underpinned by a heritable genetic component to facilitate adaptive evolution. To investigate whether the observed substantial shifts in gene expression between the two ecomorphs are associated with a heritable genetic basis, we conducted a genome-wide search of two major regulatory mechanisms, expression and splicing QTLs. From a set of 107,456 high-confidence SNPs and indels, we located 3,518 *cis*-eQTLs that demonstrated ecomorph-specific divergent regulation of 1,036 genes (5.4% of the total expressed genes; FDR-corrected *P* < 0.05; supplementary table S7, Supplementary Material online). GO analysis of *cis*-eQTL target genes revealed a large number of enriched GO terms (*n* = 144; FDR-corrected *P* < 0.05; supplementary table S8, Supplementary Material online), which are predominantly involved in DNA methylation regulation, histone modification and chromatin remodeling, suggesting a role of environmental and epigenetic mechanisms in shaping the transcriptional landscapes underpinning trophic diversification (Duncan et al. 2014). Interestingly, we also found enrichment of genes involved in musculoskeletal formation which are known to shape craniofacial phenotypes in cichlids (Gunter et al. 2013; Schneider et al. 2014; Hulsey et al. 2016; Singh et al. 2021), as well as processes involved in the regulation of cell junction assembly which are implicated in lip morphogenesis linked to foraging strategy in a Lake Tanganyika cichlid (Lecaudey et al. 2021). We further identified 4,634 *cis*-sQTLs associated with ecomorph-specific regulation of excision ratios in one or more intron clusters in 2,143 genes (11.1% of the total expressed genes; FDR-corrected *P* < 0.05; supplementary table S9, Supplementary Material online). Genes under divergent regulation from *cis*-sQTLs are enriched for GO terms related to bilateral symmetry determination, calcium pathways, and retinoic acid receptor signaling processes (*n* = 65; FDR-corrected *P* < 0.05; supplementary table S10, Supplementary Material online), which have been repeatedly implicated in LPJ plasticity networks (Gunter et al. 2013; Schneider et al. 2014; Hulsey et al. 2016). Taken together, these results demonstrate a role of eQTL and sQTL regulatory mechanisms in facilitating the diversification of complex trophic traits.

**Fig. 3.**
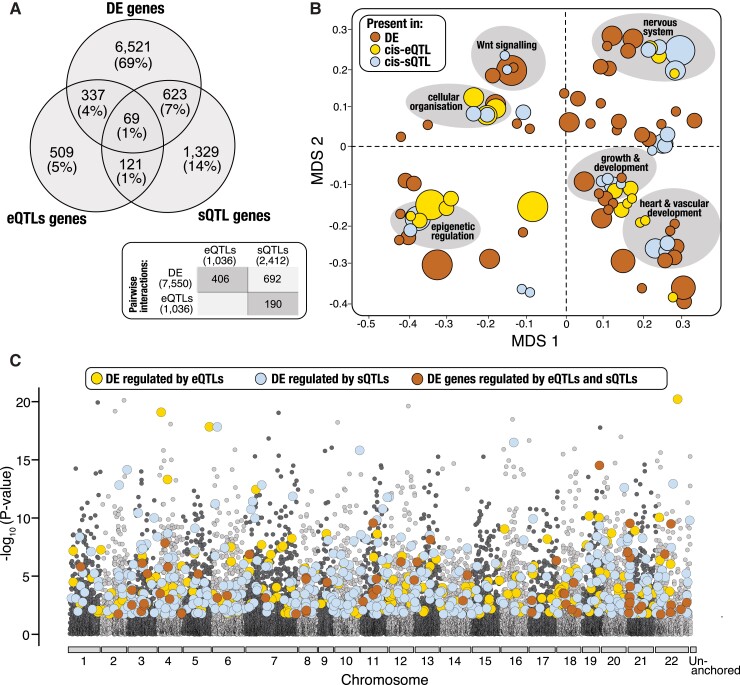
*Cis*-regulatory elements associated with expression variation between ecomorphs. (*A*) Venn diagram showing the number and proportion of genes that overlap between differential expression (DE), *cis*-regulatory splicing QTLs (*cis*-sQTL) and *cis*-regulatory expression QTLs (cis-eQTL) analyses. (*B*) Multi-dimensional scaling (MDS) plot showing enriched biological process GO terms identified for significant gene sets. Dark orange circles represent enriched terms for DE genes, yellow circles represent enriched terms for *cis*-eQTL genes, and blue circles represent enriched terms for cis-sQTL genes (FDR < 0.05). GO terms were clustered based on semantic similarity of functional classifications. The size of the circle corresponds to the number of genes associated with each GO term cluster. Descriptions are given for GO cluster functions that were shared across all three datasets (DE, *cis*-eQTL, and *cis*-sQTL genes). (*C*) Manhattan plot showing the genomic distribution of differentially expressed (DE) genes that were associated with divergent regulation from expression or splicing QTLs (according to their position in *M. zebra* reference genome). DE genes regulated by *cis*-eQTL variants are represented by yellow circles, DE genes regulated by *cis*-sQTL variants are represented by blue circles and DE genes regulated by both *cis*-eQTL and *cis*-sQTL variants are represented by dark orange circles. Chromosomes are highlighted by alternating colours and un-anchored scaffolds are located at the right end of the *x*-axis. The *y*-axis relates to the magnitude of expression divergence in individual genes, given as –log_10_ transformed FDR-corrected *P*-values generated from the global differential expression analysis (based on the total set of 19,237 expressed genes). The black dashed line indicates the 5% FDR threshold.

To quantitatively assess the relationship between expression and splicing QTLs and divergent gene expression, we searched for overlap in the ecomorph-specific gene sets identified from each analysis (i.e., *cis*-eQTL genes, *cis*-sQTL genes and DE genes). In total we found that 5.4% of DE genes are under divergent regulation from *cis*-eQTLs, representing 40% of detected eQTLs (*n* = 1,705 *cis*-eQTLs across 406 genes, Hypergeometric test: *P* = 1.064e-31; fig. 3*A* and supplementary table S11, Supplementary Material online), 9.2% of DE genes are under divergent regulation from *cis*-sQTLs, representing 65% of detected sQTLs (*n* = 3,024 cis-sQTLs across 692 genes Hypergeometric test: *P* = 7.164e-12; fig. 3*A* and supplementary table S11, Supplementary Material online), and 1% of DE genes are under divergent regulation from both expression and splicing QTLs (*n* = 69 genes; Hypergeometric test: *P* > 0.05; fig. 3*A* and supplementary table S11, Supplementary Material online). In Lake Masoko, the benthic and littoral ecomorphs exhibit very low average genomic divergence, with a small number highly differentiated genomic regions restricted to a few specific (‘islands’) of speciation (Malinsky et al. 2015). Therefore, to determine whether a similar pattern was observed for transcriptional divergence associated with overlapping gene sets we examined the genomic distribution of the 1,029 genes that were identified as having differential expression patterns mediated by one or more *cis*-regulatory QTL variants (total *cis*-eQTLs = 1,705, total *cis*-sQTLs = 3,024). We found that regions associated with extensive transcriptional divergence are more or less evenly distributed across the genome and are not conserved to specific genomic regions (fig. 3*C*). This suggests that large shifts in the transcriptional landscape are facilitated by divergent *cis*-regulatory elements during the early stages of ecological diversification.

**Fig. 4.**
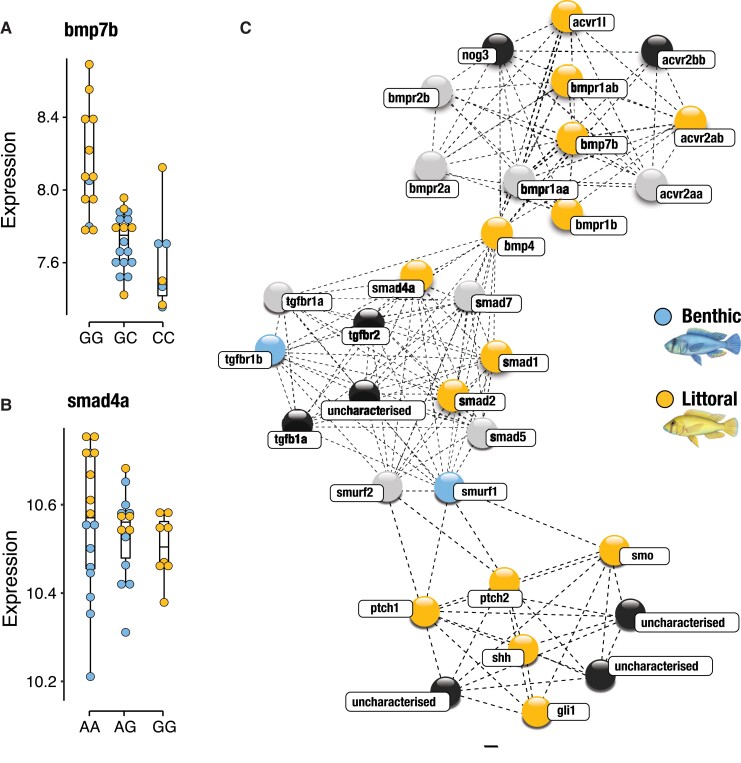
Divergent regulation in candidate genes underlying LPJ trait divergence. Associations between *cis*-eQTL genotypes and the level of expression (normalized counts) in two candidate genes involved in LPJ adaptation pathways, (*A*) *bmp7b* (Bone morphogenic protein 7*b*) within the BMP-signaling pathway and (*B*) *smad4a* (SMAD family member 4*a*) within the Hh-signaling pathway. Gene abundance per individual, per genotype is shown as blue circles for benthic individuals and yellow circles for littoral individuals. (*C*) Schematic of gene network interactions for both candidate genes. Gene interactions were deduced based on available data for *Danio rerio* and *Maylandia zebra* in STRING (https://string-db.org). Blue gene nodes represent significant upregulation in the benthic ecomorph, yellow nodes represent significant upregulation in the littoral ecomorph, grey nodes represent genes that were recovered but showed no significant differential expression or regulation between ecomorphs, and black nodes represent genes that were not recovered in our dataset.

In line with these results, we identified several differentially expressed genes under divergent regulation from expression, splicing or both QTL mechanisms that have established roles in shaping craniofacial phenotypes associated with diet specialization and feeding modality in cichlids, such as *bmp7b*, *cxcr1*, *lef1*, *smad4a*, *smurf1*, *pkp1*, *rarg*, *tnnt1*, and *usp28* (fig. 4 and Supplementary tables S6, S7 and S8, Supplementary Material online; Gunter et al. 2013; Schneider et al. 2014; Hulsey et al. 2016; Singh et al. 2017, 2021; Conith et al. 2018). Of specific interest, we identified two genes, *bmp7b* and *smad4a*, as potential candidates of LPJ adaptation in Lake Masoko. Both genes have been repeatedly linked to craniofacial adaptation in cichlids (*bmp7b* in BMP signaling and *smad4* in BMP, Wnt and TGF-b [transforming growth factor beta] signaling pathways; fig. 4 and supplementary tables S6, S7 and S8, Supplementary Material online), and more specifically to tooth patterning and regeneration in cichlids and other vertebrates (Wang et al. 2012; Hulsey et al. 2016; Yun et al. 2016; Zurowski et al. 2018; Bloomquist et al. 2019; Malik et al. 2020). For *bmp7b,* we found that gene expression level is higher in homozygous individuals for the major allele eQTL genotype, compared with both heterozygous and minor allele homozygous individuals (fig. 4). For *smad4*, minor allele homozygous individuals are exclusively associated with littorals individuals (50% of littorals and 0% of benthics are minor allele homozygous), with heterozygous genotypes more typically associated with benthic individuals compared to littorals (60% of benthics and 20% of littorals are heterozygous; fig. 4). Overall, minor allele eQTL genotypes for *bmp7b*, heterozygous allele sQTL genotypes for *smad4* and reduced expression of both genes is more typically associated with benthic individuals, which show narrower, papilliform LPJ phenotypes, with significantly smaller teeth compared with littoral individuals (figs. 1 and 4). Taken together, these results highlight both expression and splicing *cis*-acting QTLs as significant regulators of differential expression underlying complex trait evolution and provide an invaluable insight into the regulatory molecular basis of trophic niche diversification in cichlids.

### Evidence for Divergent Selection Facilitated by Large-effect *cis*-eQTLs

To further investigate the functional genomic basis and potential role of *cis*-regulatory variants in transcriptome evolution and ecological speciation we applied two approaches. First, we looked at the association between ecomorph-specific expression and splicing QTLs and genome-wide differentiation (*F*_ST_, estimated using non-overlapping 10 kb sliding window averages to avoid bias from any single highly differentiated SNPs within a given window). We found that *cis-*regulatory eQTL and sQTL variants associated with differential gene expression are significantly more likely to be located in genomic regions with higher genetic differentiation between ecomorphs (*F*_ST_ = 0.050 ± 0.0015 for eQTLs and 0.036 ± 0.0009 for sQTLs associated with DE genes; mean ± se), compared to the genome-wide average (*F*_ST_ = 0.018 ± 0.0002; mean ± se; Kruskal-Wallis: χ^2^ = 1584.4, *P* < 2.16e-16).

**Fig. 5.**
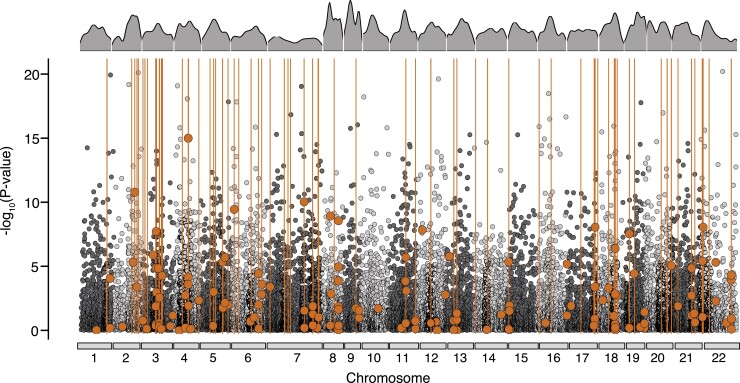
Signatures of selection. Genomic distribution of genes associated with signatures of positive selection and the associated magnitude of expression divergence, given the –log_10_ transformed FDR-corrected *P*-values generated from the global differential expression analysis across all anchored chromosomes (based on 19,237 expressed genes). Genes showing evidence of significant divergent selection between benthic and littoral ecomorphs are highlighted with dark orange points, and regions with high H12 selection scores illustrated by the orange lines. The gene density per chromosome is depicted along the top section of the plot.

Second, we determined whether patterns of ecomorph-specific divergence in gene expression, and expression and splicing QTLs are associated with genetic signatures of selection. We performed genome scans using two complementary haplotype-based statistics, xpEHH and xp-nSL to search for evidence of recent selective sweeps (Szpiech and Hernandez 2014). To determine whether genes under selection are associated with hard or soft sweeps we used a combination of H2/H1 and H12 statistics, following the approach developed by Garud et al. (2015). Surprisingly, given the very recent divergence of this species pair, we found evidence of recent selection in 640 SNPs (from a set of 107,456 high-confidence transcriptome SNPs), which are associated with 169 genes (fig. 5). Consistent with early-stage speciation, and selection on standing genetic variation (Hermisson and Pennings 2005; Przeworski et al. 2005; Feder and Nosil 2010), we identified soft sweeps as the dominant mode of adaptation in Lake Masoko *A. calliptera*, with the complete set of 640 SNPs under selection associated with evidence of soft sweeps (supplementary table S12, Supplementary Material online). Genes under selection are enriched for a total of 74 GO terms, including epithelial cell differentiation, developmental growth and morphogenesis, and neuron projection development (FDR-corrected *P*-value < 0.05; supplementary table S13, Supplementary Material online).

We found considerable overlap between genes under selection and genes that are differentially expressed (70 out of 169; Hypergeometric test: *P* = 4.917e-08), genes under divergent regulation from *cis*-eQTLs (13 out of 169; Hypergeometric test: *P* = 0.0011), and genes under divergent regulation from *cis*-sQTLs (36 out of 169; Hypergeometric test: *P* = 8.121e-11) (supplementary fig. S4 and supplementary table S11, Supplementary Material online). Moreover, we found three (out of 169) genes are shared across all four analyses (Hypergeometric test: *P* > 0.05; supplementary table S11, Supplementary Material online), which we identified as the co-stimulatory molecule CD83 and two uncharacterized serine-type endopeptidase proteins (LOC112429976 and LOC112435604). Interestingly, CD83 is involved in adaptive immune response, and specifically the activation of the major histocompatibility complex (MHC) class II which has a widely recognized role during local adaptation (Eizaguirre and Lenz 2010; Eizaguirre et al. 2012; Lenz et al. 2013a). Serine-type endopeptidase proteins are functionally linked to regulation of blood coagulation (Owen 2006), which is often associated with adaptive response to hypoxia (Gertler et al. 1991; Lee et al. 2020). Taken together, these results provide direct evidence of positive selection on regulatory transcriptional mechanisms associated with trophic diet specialization and occupation of habitats with different oxygen concentrations. They also suggest that regulatory variants underpinning transcriptional diversity are major facilitators of rapid ecological diversification.

## Discussion

In this study, we simultaneously investigated changes in two *cis*-regulatory mechanisms, expression and splicing QTLs, and their relative contribution to divergent gene expression patterns underpinning ecological speciation in cichlids. We found that substantial transcriptional modification facilitates ecological diversification, despite the absence of widespread genomic differences and ongoing gene flow (Malinsky et al. 2015). We also assessed several novel aspects of eco-morphological divergence within this species pair, including a detailed analysis of LPJ morphology (fig. 1*E*–*H*, and supplementary fig. S1, Supplementary Material online). We found that the observed direction and extent of LPJ divergence was comparable to divergence patterns in species pairs in older, more established cichlid radiations (Muschick et al. 2012; Kusche et al. 2014; Burress 2016; Hulsey et al. 2020). We also report the first evidence of niche-specific differences in parasite load, and divergent expression regulation of major adaptive immune response networks between ecomorphs in Lake Masoko (supplementary fig. S3 and supplementary tables S11 and S12, Supplementary Material online), which has emerged recently as important driver of host speciation in freshwater fishes (Blais et al. 2007; Maan et al. 2008; Matthews et al. 2010; Karvonen et al. 2013, 2015, 2018; Meyer et al. 2019). The observed divergence in multiple eco-morphological traits (fig. 1), coupled with genomic evidence of widespread recent selection, is consistent with a scenario of multifarious selection acting on numerous axes of the phenotype simultaneously.

We suggest that substantial shifts in the transcriptional landscape have allowed the rapid evolution of extensive phenotypic diversity despite a relatively low level of overall genomic differentiation between these ecomorphs. We found that a remarkable proportion of genes (39% of expressed genes, representing 24% of genes genome-wide) exhibit divergent expression between benthic and littoral ecomorphs (fig. 2*B*), which is markedly higher than has been reported previously in comparable studies of within-lake mature ecomorph pairs in natural systems (1.6% in Arctic charr (Jacobs et al. 2020; Jacobs and Elmer 2021), 9.7% in Lake whitefish (Rougeux et al. 2019), and 11.4% in European whitefish (Rougeux et al. 2019)). Gene expression responds dynamically to the environment, which can provide a flexible mechanism for rapid response to variable environmental conditions but can also contribute to long-term evolutionary responses if environmentally induced gene expression underpins traits that are favored by selection (López-Maury et al. 2008; Romero et al. 2012; Pardo-Diaz et al. 2015). We show that divergent gene expression patterns between benthic and littoral ecomorphs in Lake Masoko directly underpin several ecologically relevant phenotypes, including significant shifts in three “master-adaptation” pathways (BMP, Wnt, and Hh-signaling pathways; fig. 2*C* and supplementary tables S4, S5 and S6, Supplementary Material online) that have been repeatedly implicated in craniofacial and pharyngeal jaw adaptation to contrasting trophic environments in cichlids (Wan and Cao 2005; Nie et al. 2006; Streelman and Albertson 2006, 2008; Parsons and Albertson 2009; Gunter et al. 2013). These results strongly support a role of gene expression and regulation as a mechanism of rapid phenotypic response and divergent trophic adaptation towards the benthic and littoral habitats in Lake Masoko.

Remarkably, given the young age of our focal species pair (initial divergence estimated between 500 and 1,000 years ago (Malinsky et al. 2015)), we found that a significant proportion of differentially expressed genes are associated with changes in large-effect *cis*-regulatory variants, evidencing a genetic basis to some of the phenotypic and transcriptional diversity observed. We found that 14% of differentially expressed genes are regulated by at least one expression or splicing *cis*-regulatory QTL (fig. 3). Given that *cis* mutations are shown to be preferentially fixed by positive natural selection (Lemos et al. 2008), and that *cis*-regulatory divergence is shown to increase linearly with divergence time (Coolon et al. 2014), the high levels of *cis*-regulatory divergence observed here in such a young species pair highlight *cis*-regulatory variants as a potential mechanism driving radiation within this cichlid lineage. In line with these results, we show that target genes of expression and splicing QTLs include several genes that have widely recognized roles in shaping craniofacial phenotypes associated with diet specialization and adaptive radiation in cichlids, which includes the genes *bmp7b*, *cxcr1*, *lef1*, *smad4a*, *smurf1*, *pkp1*, *rarg*, *tnnt1*, and *usp28* (fig. 4 and supplementary tables S6, S7 and S8, Supplementary Material online; Gunter et al. 2013; Schneider et al. 2014; Hulsey et al. 2016; Singh et al. 2017, 2021; Conith et al. 2018). We identify two of these genes, *bmp7b* and *smad4a*, as potential candidates underpinning LPJ divergence between the benthic and littoral ecomorphs in Lake Masoko due to their central roles in major pathways associated with LPJ adaptation (*bmp7b* in BMP signaling and *smad4* in BMP, Wnt, and TGF-b [transforming growth factor beta] signaling pathways; fig. 4 and supplementary tables S6, S7 and S8, Supplementary Material online), and more specifically for their functional role in tooth development, patterning and regeneration in cichlids and other vertebrates (Wang et al. 2012; Hulsey et al. 2016; Yun et al. 2016; Malik et al. 2020). Therefore, we suggest that the observed transcriptional shifts in expression and regulation of these genes play a fundamental role in shaping adaptive LPJ phenotypes, facilitating trophic diet specialization (fig. 1) and subsequent ecological diversification in our focal species.

In line with this prediction, we show that *cis*-QTL variants underpinning divergence in ecologically relevant genes between ecomorphs are located within genomic regions associated with higher genetic differentiation (F_ST_ values associated with cis-QTL variants are over two-fold higher than the genome-wide average). Similar findings were recently reported in a study on sticklebacks, which showed that loci underpinning shifts in gene expression during intraspecific adaptive divergence of marine-freshwater ecotypes are predominantly regulated by *cis* mechanisms (Verta and Jones 2019), rather than *trans* mechanisms. While many studies have shown that *cis*-regulatory mechanisms contribute strongly to adaptive gene expression divergence over evolutionary long timescales (Prud’homme et al. 2006; Lemos et al. 2008; Coolon et al. 2014; He et al. 2016), these data provide the first evidence that *cis* mechanisms contribute to substantial adaptive shifts in gene expression and phenotypes over evolutionary rapid timescales (within 10–20 thousand years in sticklebacks (Verta and Jones 2019) and in less than one thousand years in our focal species). These results demonstrate a clear role of *cis*-regulatory expression and splicing QTLs in the rapid evolution of complex traits associated with ecological diversification and incipient speciation.

To further explore the role of natural selection on divergent gene expression and regulation, we tested whether *cis*-QTLs were associated with evidence of recent selective sweeps. Our results show considerable overlap of genes under divergent regulation from *cis* regulation with those that exhibit signatures of positive selection (supplementary table S11, Supplementary Material online). In support of this prediction, we found evidence of recent selection on several genes involved in epithelial cell morphogenesis and apical constriction, including the gene *iqgap2* which showed evidence of divergent regulation through sQTL variants, and has been functionally linked to tooth development in humans (Heikinheimo et al. 2015; Orlova et al. 2019). Interestingly, we also identified significant *cis*-mediated modification and selection of several genes involved in adaptive response immune system networks, including the major histocompatibility complex, and found that these were predominantly associated with adaptation towards the deep-benthic environment (supplementary tables S4 and S11, Supplementary Material online). Divergence in MHC alleles has been shown to drive host–parasite co-evolutionary dynamics within the Lake Malawi cichlid radiation (Blais et al. 2007). Given that benthic individuals exhibited an increased gill parasite load compared to littoral individuals in the current study, the observed transcriptional shifts in MHC expression and regulation could indicate that similar host–parasite co-evolutionary dynamics are ongoing in Lake Masoko. Additionally, divergence in MHC complexes have also been linked to female mate preference in three-spine sticklebacks (Matthews et al. 2010; Eizaguirre et al. 2012; Lenz et al. 2013a), meaning that such ecologically driven changes are likely to also contribute to speciation by promoting assortative mating and reproductive isolation.

These results support the hypothesis that *cis* mechanisms are potent targets of natural selection during the early stages of ecological speciation and provide novel insights into regulatory architecture underpinning rapid adaptation in natural populations. However, *cis*-regulatory variants are just one potential mechanism that can facilitate rapid evolutionary change. There are many alternative molecular mechanisms through which rapid transcriptional and phenotypic diversity can be generated that were not explored here. Specifically, microRNAs, transposable elements, and epigenetic markers are recently shown to facilitate adaptive phenotypic shifts in cichlids (Xiong et al. 2019; Carleton et al. 2020; Vernaz et al. 2022).

Overall, our results reinforce the role of gene expression in adaptive evolution. We show that substantial divergent modification of gene expression and transcriptional regulatory mechanisms underpin adaptive eco-morphological traits during early stages of sympatric speciation. These findings provide evidence that the vast transcriptional landscape acts as a rich substrate for innovation, allowing rapid diversification in resource use where resources become spatially or reliably distinct. These data suggest that an accumulation of environmentally induced transcriptional changes might promote genomic predisposition in lineages such as cichlids towards divergent adaptation and ecological speciation. Furthermore, given that our focal species and study system, *A. calliptera* from Lake Masoko, are part of the broader Lake Malawi haplochromine cichlid species flock, we suggest that our results have importance for understanding the molecular mechanisms facilitating explosive diversification in cichlids, and perhaps more broadly for large-scale adaptive radiation across a wider range of vertebrate taxa.

## Materials and Methods

### Sample Collection

*Astatotilapia calliptera* were sampled from Lake Masoko, in October 2019 (fig. 1*A*). Fish were collected using monofilament block nets and SCUBA at two habitat depths: above 5 month for littoral ecomorphs and below 20 month for benthic ecomorphs (fig. 1*B*). Benthic fish were transferred to holding containers within the lake and brought to the surface gradually over a duration of three days to allow for decompression. All fish were held in aerated containers during transportation from the field to the Tanzania Fisheries Research Institute in Kyela. A total of 113 fish were sampled, 65 from the shallow-littoral zone and 48 from the deep-benthic zone (supplementary table S1, Supplementary Material online). Fish were euthanized with MS-222 (Sigma-Aldrich), following an approved procedure. Immediately after euthanizing, fish were photographed, and standard length was measured to the nearest millimeter. Lower pharyngeal jaws (LPJs) were dissected from a subset of 38 individuals (20 littoral, 18 benthic) and stored in RNAlater. Tissues for RNA extractions were collected within five minutes of euthanasia. All individuals were preserved in ethanol and stored at −20°C for a minimum of three days before shipment to the UK. All further processing was carried out at the University of Bristol, UK.

Scientific fish collections from Lake Masoko were carried out under research permits issued by the Tanzania Commission for Science and Technology (permit number: 2019-549-NA-2019-357).

### Stable Isotope Analysis

To infer differences in feeding behavior and trophic niche specialization between ecomorphs, we performed stable isotope analysis on white muscle tissue samples from our focal fish (*n* = 113) and variety of dietary materials (*n* = 21). White muscle tissue was dissected from the left side of the ethanol-preserved fish, posterior to the operculum, above the lateral line to determine isotope signatures for carbon (δ^13^C) and nitrogen (δ^15^C). Dietary materials were represented by zooplankton, phytoplankton, macrophytes, littoral arthropods, epilithic algae, and benthic detritus (supplementary table S2, Supplementary Material online). All samples (muscle tissue and dietary materials) were oven dried at 60°C overnight. Stable isotope analyses were carried at Iso-Analytical, Crewe UK. Isotope ratios were identified using Elemental Analysis—Isotope Ratio Mass Spectrometry with a Europa Scientific 20–20 IRMS. Carbon and nitrogen isotope compositions were calibrated relative to VPDB and AIR, respectively, using IA-R042 (NBS-1577B: powdered bovine liver) as reference material. Duplicate analyses were performed on 20% of samples as a control, and instrumental accuracy was monitored using multiple reference materials (IA-R042: powered bovine liver, IA-R045/IA-R005: a mixture of ammonium sulfate and beet sugar, and IA-R046/IA-R006: a mixture of ammonium sulfate and beet sugar).

To assess overall differences in trophic in diet between benthic and littoral ecomorphs, we performed separate Kruskal-Wallis tests for carbon and nitrogen stable isotope ratios, using standard functions in R *v.*4.0.2 (R Core Team 2021). The relative proportions of potential dietary materials consumed by benthic and littoral individuals were determined using the R-package simmr (Parnell 2019), which applies a Bayesian Markov Chain Monte Carlo (MCMC) approach to estimate the composition of sources (i.e., dietary materials) that contribute to the resulting isotope signatures identified for your targets (i.e., focal species groups). The simmr MCMC model was run for 100,000 iterations with a burn rate of 1,000.

### Morphological Analysis

To quantify phenotypic variation between ecomorphs, we measured several morphological traits, including head and body shape, LPJ shape, LPJ keel depth, LPJ tooth width, and LPJ tooth length (supplementary fig. S5, Supplementary Material online).

Morphological variation in LPJs was quantified using 3D reconstructions of LPJs using micro-computed tomography (micro-CT) scans of whole specimens (*n* = 70; 40 littoral, 30 benthic). All CT data were obtained at the XTM Facility, Palaeobiology Research Group, Univesity of Bristol. A Nikon XTH225ST micro-CT scanning system was used to generate the images. Each scan used 2,750 projections and included between eight and 10 individuals. Scan resolution was determined by the size of region of interest (i.e., the head and upper section of the body), and as such was determined by the overall body size of each individual. The average voxel size across scans was approximately 23 μm. Image stacks were exported into *VG Studio v.*3.0 (Volume Graphics GmbH, 2016) and 3D models were reconstructed for each individual. We used *Avizo v.*8.0 (Hillsboro, OR) to isolate the LPJ from the rest of the skeleton and capture 2D images of the dorsal and superior-lateral perspectives. LPJ shape was analyzed using a landmark-based geometric morphometric approach, as described above. To accurately capture the shape of curved edges along the LPJ, we used a combination of standing-homologous and sliding semi-landmarks (supplementary fig. S5, Supplementary Material online). Landmark positions followed Muschick et al. (2012). Images were scaled and landmark positions were digitized with *tpsDig2 v*.2.30 (Rohlf 2004). To position sliding semi-landmarks we placed three curved lines along the outline edges of the jaw. We then fitted nine equidistant points to the line across the top outline of the jaw, and eight equidistant points to both the left and right lines. These were treated as semi-landmarks along the outline of the lower pharyngeal jaw. We the subjected the data to an iterative sliding process in tpsRelW, using a total of 10 iterations. Landmarks were then pruned to retain a final set of 8 “true” standing landmarks (orange points in supplementary fig. S5, Supplementary Material online), and 14 slid semi-landmarks (grey points in supplementary fig. S5, Supplementary Material online).

Procrustes superimposition was conducted in *MorphoJ v*.1.07a (Klingenberg 2011) to standardize landmark configuration and remove any unwanted variation related to the size, position, and orientation of the fish. Principal component analysis (PCA) was used to identify the major axes of variation in LPJ shape between littoral and benthic ecomorphs. Linear measurements for LPJ keel depth, tooth width, and tooth length were also collected using *tpsDig2*. For tooth width and length, the posterior three teeth immediately to the right side of the suture were measured (to the nearest 0.01 mm) and averaged (supplementary fig. S5, Supplementary Material online).

Head and body shape was analyzed from 2D images using a landmark-based geometric morphometric approach (*n* = 113). All individuals were photographed in a standard orientation with the head pointing to the left. Homologous landmark positions were selected based on Malinsky et al. (2015) (supplementary fig. S5, Supplementary Material online). Images were calibrated to scale, and landmarks were digitized using *tpsDig2 v*.2.30 (Rohlf 2004). Procrustes superimposition was conducted in *MorphoJ* following the same methods described for LPJ shape analyses. Principal component analysis (PCA) was used to identify the major axes of variation in head and body shape between littoral and benthic ecomorphs.

Analysis of covariance models were used to test for significant ecomorph-specific differences in all morphological traits, using body size (standard length), sex, and their interaction as covariates. Non-significant terms were excluded from final models (body size and sex were non-significant in all models).

### RNA Extraction, Sequencing, and Mapping

Total RNA was extracted from LPJ tissue using RNeasy Mini Kits (Qiagen), following the manufacturer's instructions with minor modifications. Modifications included a two-step homogenization, using a TissueLyser LT (Qiagen) followed by QIAshredder (Qiagen) column homogenization. We also performed two on-column washes with 80% ethanol immediately prior to the final RNA elution to remove any residual buffer. RNA quantity and quality were assessed using the Qubit 2.0 fluorometer (Life Technologies) with BR Assay kits and a 2100 Bioanalyser platform (Agilent), respectively. High quality RNA was achieved, with A260/280 ratios between 1.9 and 2.1 and RNA Integrity Numbers above 7. RNAseq libraries were prepared and sequenced at the Bristol Genomics Facility (University of Bristol). Separate cDNA libraries were prepared for each individual. Libraries were prepared using TruSeq Stranded mRNA Sample Preparation Kits (Illumina), in combination with a Poly-A selection step. Sequencing was performed on an Illumina NextSeq 500 platform (Illumina), using 75 bp paired-end sequencing at a sequencing depth of approximately 30 million reads per individual.

Raw reads were processed before mapping. Adapters were removed with *Scythe v*.0.9944 BETA (http://github.com/vsbuffalo/scythe/) and low-quality reads were removed with *Trimmomatic v.*0.36 (Bolger et al. 2014). *FastQC* v. 0.11.8 (Andrews 2010) was used to assess the quality of reads before and after pre-processing. Cleaned reads were then mapped to the *Maylandia zebra* reference genome (UMD2a, NCBI assembly: GCF_000238955.4; Conte et al. 2019) with *STAR v.*2.7.1a (Dobin et al. 2013). Mapping was performed using the two-pass mode in STAR in order to identify splice junctions and allow subsequent analysis of splice sites. HTSeq *v.*0.11.1 (Anders et al. 2015) was used to quantify gene expression and generate read count tables.

### Differential Gene Expression Analysis

Data were filtered to remove genes with less than 10 reads across 50% of samples, and the filtered counts were log_10_ transformed using the *rlog* function in the R-package *DESeq2 v.*1.28.1 (Love et al. 2014). We performed a PCA on the *rlog*-transformed read counts, using the R-package *PCAmethods v.*1.81.0 (Stacklies et al. 2007), to examine the overall gene expression profiles. To determine the extent of variation in gene expression between littoral and benthic ecomorphs, we conducted a differential expression (DE) analysis using negative binomial distribution models in *DESeq2*. All *P*-values were adjusted for multiple testing using Benjamini and Hochberg correction (Benjamini and Hochberg 1995) (FDR < 0.05).

### SNP Genotyping and Effect Predictions

Single nucleotide polymorphisms (SNPs) and indels were called using the physical mapping information from the *STAR* two-pass alignments of the RNAseq data, for all samples. Before calling SNPs, duplicates were marked and removed using *Picard*-*tools v*.2.20.0 (https://broadinstitute.github.io/picard/). SNPs were called using *Freebayes v*.0.9.21 (Garrison and Marth, unpublished data), specifying a minimum coverage of three reads per sample to process a site and a minimum of two reads per sample to consider an alternative allele. Using *VCFtools v*.0.1.16 (Danecek et al. 2011), we filtered the resulting SNP dataset to retain only biallelic SNPs, with a phred quality above 30, genotype quality above 30, a minor allele frequency of 10% and were present in at least 90% of all individuals. Further filtering was performed using the *vcffilter* program within *vcflib* (Garrison 2012), specifying an allele depth balance 0.30 and 0.70. This resulted in a final set of 107,456 high-confidence, gene-associated SNPs.

### *cis*-eQTL Analysis

We conducted eQTL mapping analysis to identify genetic variants putatively underlying divergent gene expression patterns between littoral and benthic ecomorphs. We focused solely on *cis*-eQTLs because we lack the substantial power required for *trans* identification in the current dataset size (*n* = 38). *Cis*-eQTLs were identified with the R-package *BootstrapQTL v*.1.0.5 (Huang et al. 2018). *BootstrapQTL* employs a three-step procedure to perform hierarchical multiple testing correction to provide more accurate effect size estimation and minimize false positives in studies, such as the current one, that have sample size constraints. We used the linear model approach within the *BootstrapQTL* package to test for gene expression variation in response to genotype variation, specifying 500 bootstraps. Genotypes for eQTL analysis were generated from the complete set of 107,456 gene-associated SNPs (called from RNAseq data) using the 012-recoding function in *VCFtools*, and normalized expression counts for the set of 19,237 expressed genes formed the expression phenotype dataset in the model, with sex specified as a covariate (gene expression ∼ genotype + sex). We defined *cis*-eQTLs as being within 1 Mb of the transcription start site of their target gene. All *P-*values were corrected for multiple testing using the three-step hierarchical correction implemented directly within *BootstrapQTL* package.

To assess the influence of population structure on cis-eQTL detection, we performed a PCA on the complete set of 107,456 SNPs using *Plink2 v*.2.00a.2.3 (Chang et al. 2015); only the first principal component was associated with ecomorph-specific genotype variation (analysis of covariance: *F*_1,36_ = 38.584, *P* = 2.598e-09; supplementary fig. S6, Supplementary Material online; *P* > 0.2 for all remaining PCs). We then performed a second model including the first principal component (PC1) as a covariate in the *BootstrapQTL* model (gene expression ∼ genotype + PC1_genotype_ + sex). Significant cis-eQTLs identified were highly consistent across both models, with 85% of detected eQTLs shared. Results for genotype corrected eQTLs are reported in supplementary table S14, Supplementary Material online.

### *cis*-sQTL Analysis

To generate splicing phenotypes for our sQTL mapping, we identified excised intron clusters with *LeafCutter v*.0.2.9 (Li et al. 2018) following the authors’ recommended pipeline. Briefly, *LeafCutter* uses bam file alignments from STAR two-pass mapping as input and generates ratios of reads supporting each alternatively excised intron. Introns with ratios < 0.001 and used in less than 40% of individuals were removed resulting in a final set of 73,311 alternatively excised intron clusters across 13,295 genes. The filtered set of intron excision ratios were quantile normalized and log_2_ transformed, and the standardized ratio values were used as the splicing phenotype for QTL mapping. *BootstrapQTL* was used to perform *cis*-sQTL mapping following the same approach described for our *cis*-eQTL mapping analysis. To test for variation in intron excision in response to genotype variation, we implemented the linear model approach in *BootstrapQTL*, using the set of 75,311 alternatively excised intron clusters as the phenotype data input and the set of 107,456 SNPs as the genotype input data.

Splicing QTLs were using a linear model as above (intron excision ratios ∼ genotype + sex). We defined *cis*-sQTLs as being within 1 Mb of their target intron cluster. All *P-*values were corrected for multiple testing using the three-step hierarchical correction implemented directly within *BootstrapQTL* package.

To assess the influence of population structure on cis-sQTL detection, we used the same approach described above for the *cis*-eQTL detection. We performed a second model including genotype PC1 as a covariate in the *BootstrapQTL* model (intron excision ratios ∼ genotype + PC1_genotype_ + sex). Significant cis-sQTLs identified were highly consistent across both sQTL models, with 94% of detected sQTLs shared. Results for genotype corrected sQTLs are reported in supplementary table S15, Supplementary Material online.

### Association Between Gene Expression Divergence and Genomic Differentiation

To determine whether differentially expressed genes under divergent regulatory control from *cis*-acting expression and splicing QTLs play a role in transcriptome evolution, we examined whether they overlap with genomic regions of high genetic differentiation (*F*_ST_). Weir and Cockerman's *F*_ST_ was calculated for the complete set of 107,456 SNPs identified from the LPJ RNAseq dataset. We used non-overlapping 10 kb sliding window averages to avoid bias from any single highly differentiated SNPs within a given window, implemented in *VCFtools*. Genome-wide window-based *F*_ST_ values were compared against averaged *F*_ST_ values for *cis*-eQTLs and *cis*-sQTLs associated with ecomorph-specific divergent regulation of differentially expressed genes using Kruskal-Wallis and subsequent pairwise Wilcoxon rank tests, performed with standard functions in R *v.*4.0.2 (R Core Team 2021).

### Signatures of Selection

We performed phasing and missing genotype imputation on the complete set of SNPs (*n* = 107,456) with *Beagle v*.4.1 (Browning and Browning 2007). Haplotype matrices and physical maps were generated for each chromosome separately using *Plink2 v*.2.00a.2.3 (Chang et al. 2015). Genome-wide signatures of selection were then identified using a combination of two complementary haplotype-based statistics to scan for evidence of recent positive selection across all anchored chromosomes (*n* = 22 chromosomes, *n* = 76,538 SNPs). Specifically, we estimated xpEHH (cross-population extended haplotype homozygosity) and xp-nSL (cross-population number of Segregating sites by Length) statistics using *selscan v*.2.0.0 (Szpiech and Hernandez 2014). To identify candidate SNPs under selection we calculated the top and bottom 1% quantiles for normalized scores of both xpEHH and xp-nSL statistics (calculated using the *norm* function within Selscan), and SNPs shared across both approaches were considered as being under selection. Finally, to infer whether SNP-linked genes under selection were associated with hard or soft sweeps we used a combination of H2/H1 and H12 statistics, using the H12_H2H1.py script from Garud et al. (2015). H2/H1 and H12 statistics were calculated for each anchored chromosome separately using overlapping windows of 25 single nucleotide variants, with a step size of 1, and allowing zero false positive single nucleotide variants per window. High H12 scores were determined using the H12 critical value, that is greater than the genome-wide median of H12. Where high H12 scores were associated with high H2/H1 ratios (>0.05), selection was considered to be influenced by soft sweeps. SNPs under selection were linked with a given gene if they were within 1 Mb of the transcription start site.

### Co-distribution of Key Regulatory Mechanisms and Patterns of Divergent Expression and Selection

We investigated the genomic co-distribution of all gene sets of interest (i.e., DE genes, *cis*-eQTL genes, *cis*-sQTL genes, and genes linked with SNPs putatively under selection). We performed hypergeometric tests with the R-package *SuperExactTest* to test the probability that genes were shared more often than expected by chance. Tests were performed using 10,000 simulations and specifying the set of 32,471 genes in the *M. zebra* reference genome as the background gene set to inform the global significance of overlap between gene sets of interest. Significance was assessed using Fisher's exact tests with an FDR correction for multiple testing (FDR < 0.05).

### Functional Enrichment Analysis

Gene ontology (GO) annotation was performed using the R-package *topGO v.*2.41.0 (Alexa and Rahnenfuhrer 2016), based on *Danio rerio* UniProt annotations. Functional enrichment analyses of GO biological process terms were identified using the *parentchild* algorithm in *topGO*. Lists of genes of interest identified by DE, *cis*-eQTL, *cis*-sQTL and selection analyses were individually compared against the reference set of 19,237 annotated genes in the *M. zebra* genome. *P*-values for GO terms were obtained using a Fisher's exact test with an FDR correction for multiple testing (FDR < 0.05). Significant GO terms were clustered based on semantic similarity using the *ViSEAGO v.*1.3.16 clustering algorithm (Brionne et al. 2019) and visualized using multi-dimensional scaling (MDS) plots. Hypergeometric tests were used to assess whether significant GO clusters associated with each gene set of interest were shared more than expected by chance, following the methods outlined above.

## Supplementary Material

msac251_Supplementary_Data

## Data Availability

RNA-sequencing raw data have been deposited in the European Nucleotide Archive (ENA) database under the project accession number PRJEB57348.
