# Supplementary material for: Ecological Speciation Promoted by Divergent Regulation of Functional Genes Within African Cichlid Fishes
Source: Mol Biol Evol. 2022 Nov 15;39(11):msac251. doi: 10.1093/molbev/msac251 (PMC10101686; doi:10.1093/molbev/msac251)
Supplement: msac251_Supplementary_Data [file msac251_supplementary_data.zip › Carruthers et al. - Supplementary Materials - Figures S1-S6 - ACCEPTED.docx]

Supplementary Materials:

**Ecological speciation promoted by divergent regulation of functional genes within African cichlid fishes**

Madeleine Carruthers*, Duncan E. Edgley, Andrew D. Saxon, Nestory P. Gabagambi, Asilatu Shechonge, Eric A. Miska, Richard Durbin, Jon R. Bridle, George F. Turner & Martin J. Genner

*Corresponding author: Email: [ph19872@bristol.ac.uk](mailto:ph19872@bristol.ac.uk)

**This PDF file includes:**

Figs. S1 to S6

**Other Supplementary Materials for this manuscript include the following:**

Tables S1 to S15

**Supplementary Figures**

**Supplementary Figure 1. Major axes of LPJ shape change. a)** LPJ shape change along PC1. **b)** LPJ shape change along PC2. **c)** LPJ shape change along PC3. The percent of expression variation explained by each axis is given in parentheses. Outline shapes represent axis extremes for all PCs. Asterisks denote significant differences between benthic (blue) and littoral (yellow) ecomorphs. Number of asterisks represents the level of significance. N.S represents non-significant differences between ecotypes. N = 70.

**Supplementary Figure 2. Major axes of body shape change. a)** Body shape changes along PC1. **b)** Body shape changes along PC2. **c)** Body shape changes along PC3. The percent of expression variation explained by each axis is given in parentheses. Outline shapes represent axis extremes for all PCs. Asterisks denote significant differences between benthic (blue) and littoral (yellow) ecomorphs. Number of asterisks represents the level of significance. N.S represents non-significant differences between ecomorphs. N = 113.

**Supplementary Figure 3. Parasite load differences.** Difference in parasite loads of the gill ectoparasite species, *Lamproglena monodi* identified for benthic (blue) and littoral (yellow) ecomorphs. Grey points represent them mean values for each ecomorphs. Asterisks denote significant differences between benthic and littoral ecomorphs. Number of asterisks represents the level of significance. N = 38.

**Supplementary Figure 4. Frequency of shared genes between transcriptional analyses.** Different analyses are indicated by the dots for DE (differentially expressed genes, n = 7,550), eQTL (*cis* expression QTL regulated genes; n= 1,036), eQTL (*cis* splicing QTL regulated genes; n= 2,143), and SELECTION (genes under selection; n = 169). Individual dots represent the number of genes unique to a given analysis. Linked dots represent the number of shared genes across analyses.

**Supplementary Figure 5. Schematic of morphological measurements for LPJ and body shape. a)** Dorsal view of the LPJ. Landmark scheme used for geometric morphometric analysis of LPJ shape. Orange points represent standing landmark positions and grey points represent sliding semi-landmarks; total of 22 landmarks. LPJ tooth width measurements were collected from the first three posterior teeth immediately to the right of the suture line and are highlighted in red. **b)** Posterior view of the LPJ. Keel depth was measured following the suture line (dashed line). The first three teeth to the right (from a dorsal perspective) were used for tooth length measurements and are highlighted in red. **c)** Landmark scheme used for geometric morphometric analysis of body shape; total of 22 landmarks.

**Supplementary Figure 6. Principal component plots of individual genotypes.** PCAs shows variation in individuals genotypes (n=38 individuals) along the first and second principal components **(a)**, and the first and third principal components **(b),** based on set of 107,456 high-confidence SNPs and indels identified across the transcriptome.

**Supplementary Tables**

Supplementary tables (S1-S15) are provided in a single separate file.
